# Supplementary material for: Effects of gastrocnemius functional massage on lower extemity spasticity, spatio- temporal gait variables and fall risk in patients with stroke: A randomized controlled trial
Source: PLoS One. 2025 Sep 24;20(9):e0332308. doi: 10.1371/journal.pone.0332308 (PMC12459846; doi:10.1371/journal.pone.0332308)
Supplement: S2 Text — (DOCX) [file pone.0332308.s002.docx]

**PAMUKKALE UNIVERSITY**

**NON-INTERVENTIONAL CLINICAL RESEARCH ETHICS COMMITTEE**

**APPLICATION FORM**

**Date:** 12/12/2023

**Title of the Research:** " Effects of Gastrocnemius Function Massage on Lower Extemity Spasticity, Spatio- Temporal Gait Variables and Fall Risk in Patients with Stroke: A Randomized Controlled Trial"

**Research Team**

**Principal Investigator:**

**Name-Surname:** Emre BASKAN

- **Field of Expertise-Title:** Physiotherapy and Rehabilitation - Associate Professor
- **Institution/City:** Pamukkale University, Faculty of Physical Therapy and Rehabilitation / DENIZLI
- **Address:** Pamukkale University, Faculty of Physical Therapy and Rehabilitation
- **Role in the Project:** Principal Investigator

**Other Researchers**

**Name-Surname:** Aziz DENGIZ

- **Field of Expertise-Title:** Physiotherapy and Rehabilitation - Assistant Professor
- **Institution/City:** Mus Alparslan University, Faculty of Health Sciences, Department of Physiotherapy and Rehabilitation
- **Address:** Mus Alparslan University, Faculty of Health Sciences
- **Role in the Project:** Data Collector
- **Email:** ptazizdengiz@gmail.com

**Name-Surname:** Güzin KARA

- **Field of Expertise-Title:** Physiotherapy and Rehabilitation - Assistant Professor
- **Institution/City:** Pamukkale University, Faculty of Physical Therapy and Rehabilitation / DENIZLI
- **Address:** Pamukkale University, Faculty of Physical Therapy and Rehabilitation
- **Role in the Project:** Data Collector

**Name-Surname:** Serbay SEKEROZ

- **Field of Expertise-Title:** Physiotherapy and Rehabilitation - Doctor of Physiotherapy
- **Institution/City:** Van Yüzüncü Yıl University, Faculty of Health Sciences, Department of Physiotherapy and Rehabilitation
- **Address:** Van Yüzüncü Yıl University, Faculty of Health Sciences
- **Role in the Project:** Data Collector
- **Email:** serbaysekeroz@gmail.com

**Nature of the Research**

**Reason for the Research**

- Specialty Thesis Study
- Doctoral Thesis Study
- Graduate Thesis Study
- **X** Other (Clinical Study)

**Type of Research**

**I. Epidemiological**

- Descriptive
  - Case Series
  - Cross-Sectional

**II. Clinical Research**

- Open Uncontrolled Study
- **X** Controlled Randomized
- **X** Parallel Groups
- Crossover Groups
- Placebo-Controlled
- Single-Blind
- Double-Blind
- Other

**III. Survey**
**IV. Archive**
**V. Other Types of Research**

**Research Location**

- **X** Hospital
- Outpatient Clinic
- Health Center
- Field
- Others

**Justification and Purpose of the Research**

Stroke is the most common life-threatening neurological disease, ranking third as a cause of death and first in morbidity. Hemiplegia/hemiparesis and motor limitations observed after cerebrovascular events (CVE) include numerous problems such as loss of awareness of one side of the body, disturbances in body image, and visual impairments, negatively affecting sensory and perceptual functions. These issues cause difficulties in activities such as bathing, personal care, mobility inside and outside the home, and transfer activities (bed to wheelchair, bathroom, toilet, etc.). Consequently, these problems significantly impact vital functions such as walking, running, and climbing stairs, reducing the quality of life and limiting participation in daily activities.

Neurological problems play a crucial role in issues related to quality of life, activity participation, and mobility. Another significant issue for stroke survivors to maintain functional lives is muscular problems. The skeletal muscle is one of the most adaptable structures in the human body. Almost every muscle's structural aspects (architecture, genetic expression, fibril distribution, number and distribution of alpha motor units, motor end plates, number of sarcomeres, etc.) have the potential to change with appropriate stimulation.

In stroke patients, spasticity, loss of joint range of motion, sensory problems, and shortening of ligaments and muscles have been found to cause sarcomere loss in immobilized muscles in shortened positions. Additionally, the remaining sarcomeres lengthen to increase tension in the shortened position. The immobilization of hypertonic muscles in a shortened position creates a potential for contracture due to muscle atrophy, sarcomere loss, weakness in actin-myosin cross-bridges, and connective tissue accumulation.

To eliminate neurological deficits and the resulting disabilities and to facilitate coordinated, voluntary, functional movements, neuromuscular plasticity must be established. Manual therapy, a widely used rehabilitation method, serves this purpose effectively.

Manual therapy, which has been applied for over a century for treating pain and functional disorders in the spine and extremity joints, includes techniques such as manipulation, mobilization, and post-isometric relaxation. It aims to restore movement in restricted joints, reduce pain, and maintain body mechanics. The safe and effective application of manual therapy requires a detailed assessment of the locomotor system's anatomy, biomechanics, and neurophysiology.

Recent studies have shown that manual therapy has a reducing effect on spasticity, a common and significant complication in stroke patients. Studies have also reported that continuous distraction or spinal manipulation can stimulate type 3 mechanoreceptors in joints, potentially leading to muscle inhibition. Based on these findings, this study aims to investigate the efficacy of manual therapy on the lower extremities of stroke patients, which significantly affects life functions.

**Materials and Methods**

**Participants**

A minimum of 26 stroke patients aged 30 years and older, receiving treatment at Pamukkale University Adult Neurological Rehabilitation Unit (Outpatient), with no additional neurological disorders or orthopedic, communication, or mental disabilities affecting assessments, and who voluntarily consent to participate will be included.

Personal and disease-related data will be collected using a demographic information form, gait parameters will be evaluated using the LEGsys gait analysis system, functional mobility using the Timed Up and Go Test, and spasticity using the Modified Ashworth Scale. **Participation in the study will be based on voluntariness. Evaluations will be conducted through face-to-face interviews with participants. Evaluations will be performed before treatment, after treatment, and three months post-treatment.**

Patients included in the study will be randomized into two groups: the control group will receive one hour of conventional physiotherapy and sham functional massage, while the treatment group will receive conventional physiotherapy in addition to functional gastrocnemius massage twice a week for 10 minutes over a six-week period.

### ****Surveys and Scales to be Applied to Cases:****

#### ****Sociodemographic Data Evaluation Form:****

Information such as gender, age, stroke type, and assistive devices used by the cases will be recorded in the prepared sociodemographic data form.

#### ****Spatio-Temporal Gait Analysis (LEGsys) and Timed Up and Go Test****

The gait performance of the cases will be evaluated using the LEGsys spatio-temporal gait analysis device developed by BioSensics. The device consists of two sensors. The sensors will be placed with Velcro between the ankle and knee joint of the patient, closer to the ankle. The device is controlled via dedicated software on a computer and transmits the collected raw data in real-time to the computer via Bluetooth. The software analyzes the raw data received from the device and converts them into results. The Modified Timed Up and Go Test (MTUG), supported by the device, will be used for evaluation. The test will be repeated twice, and the average duration will be recorded. LEGsys provides information on double-step length, duration, speed, standing up, turning, and sitting times, as well as the total duration of walking.

#### ****Modified Ashworth Scale****

The Ashworth Scale, originally defined by Ashworth to score spastic extremities based on the resistance encountered during passive movement (on a scale of 0-4), was modified by Pedersen with the addition of a 1+ score. In 1987, Bohannon and colleagues further revised the Ashworth Scale to a 0-5 scoring system.

Modified Ashworth Scale:

- **0:** No increase in muscle tone.
- **1:** Slight increase in muscle tone; minimal resistance at the end of the range of motion during flexion or extension.
- **2:** Slight increase in muscle tone; resistance appears in the latter half of the range of motion, but the limb is still easily moved.
- **3:** More marked increase in muscle tone; resistance is encountered throughout most of the range of motion, but movement is still completed.
- **4:** Considerable increase in muscle tone; passive movement is difficult.
- **5:** The affected limb is rigid in flexion and extension.

Despite being subjective, MAS is a manual method that does not require any equipment and is easy to apply for the assessment of spasticity.

**Statistical Analysis**

A power analysis determined that including at least 26 participants (13 per group) in the study would achieve 90% power at a 95% confidence level. Data will be analyzed using the SPSS statistical software package. Continuous variables will be presented as mean ± standard deviation, while categorical variables will be expressed as count and percentage.

If parametric test assumptions are met, the Independent Samples T-Test will be used to compare differences between independent groups. If parametric test assumptions are not met, the Mann-Whitney U Test will be used for independent group comparisons. For dependent group comparisons, the Paired Samples T-Test will be applied if parametric test assumptions are met, while the Wilcoxon Test will be used if they are not met. Additionally, relationships between continuous variables will be analyzed using Spearman or Pearson correlation analyses, and differences between categorical variables will be examined using the Chi-Square test.

**Research Implementation Site(s):**

Pamukkale University Hospitals, Adult Neurological Rehabilitation (Outpatient) Unit

**Inclusion Criteria for Volunteers:**

Study Group: Participants aged over 30 years, with no other neurological impairment or orthopedic, mental, or communication problems that would interfere with assessments, who can stand unaided or with assistive devices for at least one minute, with a spasticity level of 3 or below according to the Modified Ashworth Scale, and who voluntarily consent to participate in the study will be included.

Control Group: The inclusion criteria are the same as those for the study group.

**Exclusion Criteria for Volunteers:**

Study Group: Participants with communication problems, outside the specified age range, with another neurological diagnosis, with orthopedic, mental, or communication problems that interfere with assessments, who cannot stand unaided or with an assistive device for one minute, with a spasticity level of 4 or above according to the Modified Ashworth Scale, and those unwilling to participate in the study will be excluded.

Control Group: The exclusion criteria are the same as those for the study group.

**Criteria for Withdrawal from the Study:** Volunteers who are unable to complete the prescribed treatments will be withdrawn from the study.

**Criteria for Study Termination:** The study will be completed once the planned number of participants is reached.

**Estimated Study Duration:**

- **Start Date:** After Ethics Committee Approval
- **End Date:** October 2024
