# Supplementary material for: Effects of gastrocnemius functional massage on lower extemity spasticity, spatio- temporal gait variables and fall risk in patients with stroke: A randomized controlled trial
Source: PLoS One. 2025 Sep 24;20(9):e0332308. doi: 10.1371/journal.pone.0332308 (PMC12459846; doi:10.1371/journal.pone.0332308)
Supplement: S3 Text — (DOCX) [file pone.0332308.s003.docx]

**PAMUKKALE ÜNİVERSİTESİ**

**GİRİŞİMSEL OLMAYAN KLİNİK ARAŞTIRMALAR ETİK KURULU**

**BAŞVURU FORMU**

Tarih:12 /12/2023

# Araştırmanın Adı: “İnmeli Bireylerde Hemiplejik Alt Ekstremiteye Uygulanan Manuel Terapinin Etkilerinin İncelenmesi’’

**Araştırma Ekibi:**

1. **Sorumlu Araştırıcı:**

Adı-soyadı : Emre BASKAN

Uzmanlık alanı-Ünvanı : Fizyoterapi ve Rehabilitasyon- Doç Dr.

Çalıştığı Kurum/Şehir : Pamukkale Üniversitesi Fizik Tedavi ve Rehabilitasyon Fakültesi /DENİZLİ

Adresi : Pamukkale Üniversitesi Fizik Tedavi ve Rehabilitasyon Fakültesi

Projedeki Sorumluluğu : Sorumlu araştırıcı

1. **Diğer araştırmacılar**

Adı-soyadı : AZİZ DENGİZ

Uzmanlık alanı- Ünvanı : Fizyoterapi ve Rehabilitasyon – Dr. Öğr. Üyesi

Çalıştığı Kurum/Şehir : Muş Alparslan Üniversitesi, Sağlık Bilimleri Fakültesi, Fizyoterapi ve Rehabilitasyon Bölümü

Adresi : Muş Alparslan Üniversitesi, Sağlık Bilimleri Fakültesi.

Projedeki Sorumluluğu : Veri toplayıcısı

e-posta : ptazizdengiz@gmail.com

Adı-soyadı : Güzin KARA

Uzmanlık alanı-Ünvanı : Fizyoterapi ve Rehabilitasyon- Dr. Öğr. Üyesi

Çalıştığı Kurum/Şehir : Pamukkale Üniversitesi Fizik Tedavi ve Rehabilitasyon Fakültesi /DENİZLİ

Adresi : Pamukkale Üniversitesi Fizik Tedavi ve Rehabilitasyon Fakültesi

Projedeki Sorumluluğu : Veri toplayıcısı

Adı-soyadı :Serbay Şekeröz

Uzmanlık alanı- Ünvanı : Fizyoterapi ve Rehabilitasyon – Dr. Fzt.

Çalıştığı Kurum/Şehir :Van Yüzüncü yıl Üniversitesi, Sağlık Bilimleri Fakültesi, Fizyoterapi ve Rehabilitasyon Bölümü

Adresi : Van Yüzüncü yıl Üniversitesi, Sağlık Bilimleri Fakültesi.

Projedeki Sorumluluğu : Veri toplayıcısı

e-posta : serbaysekeroz@gmail.com

**Araştırmanın nedeni**

Uzmanlık tez çalışması

Doktora tez çalışması

Lisansüstü tez çalışması

Diğer (Klinik çalışma)

**Araştırmanın niteliği**

**I. Epidemiyolojik**

Tanımlayıcı

- Olgu serisi
- Kesitsel

**II. Klinik araştırma**

Açık kontrolsüz çalışma

Kontrollü randomize

Paralel gruplar

Çapraz gruplar

Plasebo kontrollu

Tek kör

Çift kör

Diğer

**III. Anket**

**IV. Arşiv**

**V. Diğer tür araştırmalar**

**Araştırmanın yapılacağı yer**

Hastane

Poliklinik

Sağlık ocağı

Saha

Diğerleri

# Araştırmanın Gerekçesi ve Amacı:

# İnme yaşamı tehdit eden en sık nörolojik hastalık olup, ölüm nedeni olarak 3. sırada, morbidite açısından da 1. sırada yer almaktadır. SVO sonrası görülen hemipleji/hemiparezi ve motor kısıtlanmaların yanı sıra vücudun bir tarafının farkında olma ve vücut imajında kayıp, görsel bozukluklar gibi pek çok problemi kapsayan; duyusal ve algısal fonksiyonlarda olumsuz etkilere neden olan bir hastalık durumudur. Bu yüzden banyo yapma, kişisel bakım aktiviteleri, ev içi ve dışında mobiliteyi sürdürme, transfer aktiviteleri (yataktan, tekerlekli sandalyeye, banyoya, tuvalete) gibi aktivitelerde problemlerle karşılaşılmaktadır. Sonuç olarak bu problemler inmede; yürüme, koşma, merdiven çıkma gibi hareket edebilme becerileri ve günlük yaşam aktivitelerini sürdürme kapasitesi gibi yaşamsal fonksiyonları etkileyerek, bireyin yaşam kalitesini olumsuz etkilemektedir ve günlük yaşam aktivitelerine katılımını önemli derecede kısıtlamaktadır.

Yaşam kalitesi, aktivite katılımı ve mobilitede görülen problemler arasında nörolojik sorunlar önemli bir yer tutarken, inme geçirmiş bireylerin yaşamlarını fonksiyonel olarak sürdürebilmeleri için bir diğer önemli başlık ise kassal sorunlardır.

İskelet kası, insan vücudunun en şekil verilebilir yapılarından biridir. Hemen hemen her kasın yapısal yönü (mimari, genetik ifadesi, fibril dağılımı, alfa motor ünitlerin sayı ve dağılımı, motor son plak, sarkomer sayısı gibi) uygun bir stimülusla değişim potansiyeline sahiptir. Kas fibrilleri yapısal olarak çeşitliliğe sahiptir ve bu çeşitlilik insan hareketini desteklemek için ihtiyaç duyulan çeşitli rol ve fonksiyonlara izin verir.

İnmeli hastalarda spastisite, eklem hareket açıklığı kaybı, duyu problemleri, ligamentlerin ve kasların kısalması gibi sorunlar sebebiyle kısalmış pozisyonda immobilize olan kaslarda sarkomer kaybı bulunmuştur. Kalan sarkomerlerin de kısalmış pozisyondaki gerilimi arttırmak için uzunluğunu arttırdığı bulunmuştur. Ayrıca, hipertonik kasların kısalmış pozisyonundaki immobilizasyonu kas atrofisi, sarkomer kaybı, aktin miyozin çapraz köprülerinin zayıflığı, konnektif doku birikimi ile kontraktür gelişimi için potansiyel oluşturur. Kas uzunluğu ve kuvvet uyumundaki kas dengesizliği seçici hareket kontrolü için koordinasyonun tamamını etkileyecektir. Fonksiyonu bozan değişikliklerle birlikte temel sorun, kas uzunluğunda azalma, kas sertliğinde artma olarak tanımlanmıştır ve bunlar zayıf fonksiyonel durum ile görülen ikincil muskuloskeletal komplikasyonlardır.

Nörolojik lezyonlar ve bunun sonucunda ortaya çıkan ortaya çıkan yetersizliklerin ortadan kaldırılması ve koordine, istemli, kaliteli, fonksiyonel hareketin açığa çıkartılabilmesi için nöroplastisite ile kassal plastisitenin de oluşturulabilmesi gerekmektedir. Bu amaçla uygulanan manuel terapi, rehabilitasyonda kullanılan etkili bir tedavi yöntemidir.

Omurga ve ekstremite eklemlerindeki ağrı ve fonksiyon bozukluklarının tedavisinde yüz yıldan fazla bir süredir uygulanan manuel terapi, manipülasyon, mobilizasyon ve postizometrik relaksasyon derin friksiyon teknikleri gibi yöntemleri içerir. Manuel terapinin amacı, eklemlerdeki blokaj olarak adlandırılan kısıtlanmış hareketi postüral denge içinde, en yüksek derecede ve ağrısız artırıp fonksiyonu sağlamak ve beden mekaniğini korumaktır. Manuel terapinin etkin ve güvenli uygulanması için, lokomotor sistemin anatomik, biyomekanik ve nörofizyolojik olarak detaylı degerlendirilmesi gerekir.

Manuel terapi, özellikle ortopedi, nöroloji ve romatoloji alanlarındaki medikal ve rehabilitatif tedavilere katkıda bulunan manipülasyon ve mobilizasyon gibi teknikleri kapsar.

Son yıllarda yapılan çalışmalarda manuel terapinin inmeli hastalarda en çok karşılaştığımız ve önemli komplikasyonlara neden olan spastiste üzerinde azaltıcı bir etki gösterdiği ortaya konmuştur. Bir çalışmada spastik kasların bulunduğu bölgeye uygulanan spinal manüplatif itme sırasındaki EMG kayıtları, bazı hastalarda kasın gevşediğini ve o andaki EMG aktivitesinin ortadadan kalktığını göstermektedir.

Başka bir çalışmada eklemlerde bulunan tip 3 mekanoreseptörler sürekli distraksiyon veya spinal manipulasyon ile uyarılabilir ve kas inhibisyonuna neden olabilir şeklinde sonuçlar rapor edilmiştir. Bütün bu çalışmalarda yola çıkılarak yapılması planlanan çalışmada manuel terapinin inmeli bireylerde yaşam fonksiyonlarını önemli derecede etkileyen, etkilenen alt ekstremite üzerinde etkinliğininin araştırtırılması planlanmaktadır.

# Araştırmanın Gereç ve Yöntemi:

***Katılımcılar:***

Pamukkale Üniversitesi Erişkin Nörolojik Rehabilitasyon Ünitesi (Ayaktan) tarafından takip ve tedavisi yürütülen 30 yaş üstü inmeli, başka bir nörolojik özrü ve değerlendirmeleri etkileyecek ortopedik, iletişim ve mental yetersizlik problemi olmayan ve çalışmaya katılmaya gönüllü en az 26 inmeli erişkin birey çalışmaya dahil edilecektir.

Katılımcıların kişisel ve hastalıkla ilişkili bilgileri sosyodemografik verileri demografik bilgi formuyla, yürüme parametreleri LEGsys yürüme analiz sistemiyle, fonksiyonel mobiliteleri Zamanlı-kalk yürü testiyle ve spastisteleri modifiye Ashworth skalasıyla değerlendirilecektir. Araştırmaya dahil edilme gönüllülük esasına dayanacaktır. Değerlendirmeler katılımcılarla yüz yüze görüşülerek yapılacaktır. Değerlendirme tedavi öncesi, tedavi sonrası ve tedaviden 3 ay sonra yapılacaktır.

Çalışmaya dahil edilen hastalar randomizasyon sonucu 2 grubu ayrılacak kontrol grubuna 1 saat boyunca konvansiyonel fizyoterapi ve sham fonksiyon masajı uygulanırken, tedavi grubuna konvansiyonel fizyoterapiye ek olarak 6 hafta boyunca haftada 2 kez 10 dakika gastrocnemius fonksiyon masajı uygulanacaktır.

**Olgulara Uygulanacak Anket ve Ölçekler :**

- **Sosyodemografik Verileri Değerlendirme Formu:**

Olguların cinsiyet, yaş, inme tipi, kullandığı yardımcı cihazlar gibi bilgileri hazırlanan sosyodemografik veri formuna kaydedilecektir.

**Spatio-Temporal Yürüme Analizi (LEGSystm)** ve Zamanlı Kalk Yürü Testi

Olguların yürüme performansı, BioSensicstm firmasının geliştirdiği LEGSystm isimli spatio-temporal yürüme analizi cihazı ile değerlendirilmiştir. Cihaz iki adet sensörden ibarettir.Sensörler, test edilecek olgunun ayak bileği eklemi ile 18 diz eklemi arasına, ayak bileğine daha yakın olacak şekilde velkro yardımıyla yerleştirilir. Cihaz, kendisine ait yazılımı ile bilgisayardan kontrol edilir ve topladığı ham verileri anlık olarak Bluetooth aracılığı ile bilgisayara gönderir. Yazılım, cihazdan aldığı ham verileri analiz ederek sonuçlara dönüştürür. Değerlendirme için cihazın da desteklediği Modifiye Kalk ve Yürü Testi (MKYT) kullanılacaktır. Test 2 kez tekrarlanacak ve ortalama süre kaydedilecek. Legsystm, yürümenin çift adım uzunluğu, süresi ve hızı ile ayağa kalkma, dönme, oturma süreleri ile toplam süre hakkında bilgi verir.

- **Modifiye Ashworth Skalası**

Ashworth tarafından tanımlanan ve spastik ekstremitenin pasif hareketi sırasında gösterdiği dirence göre 0-4 arası puanlama yapılan bu skalaya Pedersen 1+ değerini ekleyerek modifiye etmiştir. 1987‟de ise Bohannon ve ark. Ashworth skalasını 0-5 arası puanlarla yeniden modifiye etmiştir. Modifiye Ashworth Skalası: 0: Tonus artışı yok, 1: Kas tonusunda hafif artış, ekstremite fleksiyon ya da ekstansiyona hareket ettirildiğinde hareket sonunda minimal direnç, 2: Kas tonusunda hafif artış, hareket açıklığının son yarısında ortaya çıkar ancak ekstremite kolayca hareket ettirilir, 3: Kas tonusunda daha belirgin artış, hareket alanının büyük bir kısmında ortaya çıkar, ancak hareket tamamlanır, 4: Kas tonusunda kayda değer artış, ancak pasif hareket zordur, 5: Etkilenen ekstremite fleksiyon ve ekstansiyonda rijit. MAS subjektif olmasına rağmen spastisitenin değerlendirilmesinde herhangi bir araç gerektirmeyen ve kolay uygulanabilen manuel bir yöntemdir.

**İstatistiksel Analiz**

Yapılan güç analizi sonucunda çalışmaya en az 26 kişi (her grup için 13 kişi) alındığında %95 güvenle %90 güç elde edilebileceği hesaplanmıştır. Veriler SPSS paket programıyla analiz edilecektir. Sürekli değişkenler ortalama ± standart sapma ve kategorik değişkenler sayı ve yüzde olarak verilecektir. Parametrik test varsayımları sağlandığında bağımsız grup farklılıkların karşılaştırılmasında İki Ortalama Arasındaki Farkın Önemlilik Testi; parametrik test varsayımları sağlanmadığında ise bağımsız grup farklılıkların karşılaştırılmasında Mann-Whitney U testi kullanılacaktır. Bağımlı grup karşılaştırmalarında, parametrik test varsayımları sağlandığında Paired Samples T Test; parametrik test varsayımları sağlanmadığında ise Wilcoxon Testi kullanılacaktır. Ayrıca sürekli değişkenlerin arasındaki ilişkiler Spearman ya da Pearson korelasyon analizleriyle ve kategorik değişkenler arasındaki farklılıklar ise ki kare analizi ile incelenecektir.

# Araştırmanın Uygulama Yeri/Yerleri:

Pamukkale Üniversitesi Hastaneleri Erişkin Nörolojik Rehabilitasyon (Ayaktan) Ünitesi

**Gönüllüler İçin Araştırmaya Dahil Olma Kriterleri:**

# Çalışma grubu: 30 yaş üstü, başka bir nörolojik özrü ya da değerlendirmeleri engelleyecek ortopedik, mental ve iletişim problemi bulunmayan, kendine yardım aleti ile ya da bağımsız olarak en az 1 dakika ayakta durabilen, modifiye ashworth skalasına göre spastistesi 3 ve altında olan, çalışmaya katılmaya gönüllü katılımcılar dahil edilecektir.

Kontrol grubu: Çalışma grubunun araştırmaya dahil olma kriterleriyle aynıdır.

**Gönüllüler İçin Dışlama Kriterleri:**

# Çalışma grubu: İletişim problemi olan, belirlenen yaş dağılımlarının dışında, başka nörolojik tanısı olan ve değerlendirmeleri engelleyecek şekilde ortopedik, mental ve iletişim problemi olan, kendine yardım aleti ile ya da bağımsız olarak 1 dakika ayakta duramayan, modifiye ashworth skalasına göre spastistesi 4 ve üstünde olan çalışmaya katılmaya rızası olmayan katılımcılar çalışmaya dahil edilmeyecektir.

Kontrol grubu: Çalışma grubunun dışlanma kriterleriyle aynıdır.

**Gönüllüler İçin Çalışmadan Çıkarılma Kriterleri:**

Uygulanacak tedavileri tamamlayamaması durumunda gönüllüler çalışmadan çıkarılacaktır.

**Araştırmaya son verme kriterleri:**

Planlanan olgu sayısına ulaşıldığında araştırma tamamlanacaktır.

**Araştırmanın başlama tarihi ve öngörülen süresi:**

Başlangıç Tarihi: Etik kurul onayından sonra

Bitiş: Ekim 2024
