# Supplementary material for: Effects of gastrocnemius functional massage on lower extemity spasticity, spatio- temporal gait variables and fall risk in patients with stroke: A randomized controlled trial
Source: PLoS One. 2025 Sep 24;20(9):e0332308. doi: 10.1371/journal.pone.0332308 (PMC12459846; doi:10.1371/journal.pone.0332308)
Supplement: Attachment — Submitted filename: Effects of Gastrocnemius Function Massage.docx [file pone.0332308.s005.docx]

1. It would be useful to include some important demographic details in this section, such as gender distribution, stroke etiology (ischemic/hemorrhagic), and affected side (right/left).
2. **GFM Definition:** The detailed description of the Gastrocnemius Function Massage (GFM) application is commendable. However, some expressions (for example: “the therapist gently pulls the arm and trunk backwards until the foot and gastrocnemius muscle relaxes”) need clarification in terms of anatomy and biomechanics. Do the terms “arm” and “trunk” here refer to the therapist’s or the patient’s trunk? Unclear.
3. **Sham GFM:** The “sham” method applied to the control group provides a valid comparison, but the extent to which the procedure was effective in terms of blinding (e.g., whether participants guessed their group) is not specified.
4. **Gap:** The fact that NDT was administered to all participants suggests that GFM was the only variable; however, more information on session length and degree of individualization in content for NDT would be helpful.
5. **MAS:** The explanations are sufficient, but it is not stated how many different assessors made the assessments and whether the assessors were blinded.
6. **Gait Analysis - Legsystm:** Adequate technical explanation is given. However, it would have been appropriate to refer to the validity and reliability information of the device.
7. **Deficiency:** Taking the effect size as 1 is a very high assumption. It should be stated which source or preliminary study this value is based on.
8. Sentence structures should be simplified; especially the part explaining the GFM application should be written in clearer and more technical terms.
9. A reference to the effect size used in the sample size calculation should be provided.
10. It should be stated whether the person(s) performing the spasticity assessment are the same person and whether blindness is ensured.
11. There are grammar and fluency problems in some sentences. Example: In the sentence “Participants in both groups had similar in terms of…” the expression “had similar” is used incompletely. The correct usage should be “were similar in terms of…”
