# Supplementary material for: Effects of gastrocnemius functional massage on lower extemity spasticity, spatio- temporal gait variables and fall risk in patients with stroke: A randomized controlled trial
Source: PLoS One. 2025 Sep 24;20(9):e0332308. doi: 10.1371/journal.pone.0332308 (PMC12459846; doi:10.1371/journal.pone.0332308)
Supplement: Attachment — Submitted filename: Response to Reviewers.docx [file pone.0332308.s006.docx]

Dear Editor,

First of all, we would like to thank you and the valuable reviewers for your time and interest in helping to improve the article. Revisions were made meticulously based on the suggestions of the reviewers. Revisions are highlighted in the text and explanations are provided below.
Sincerely.

**Reviewer #1:**

Dear Reviewer,

Thank you very much for your valuable and constructive comments. They have helped us improve the transparency and scientific rigor of our manuscript. Below we provide point-by-point responses describing the changes made to the text.

1. Sample size and power analysis details

Reviewer comment:

“Based on an expected effect size of 1 ... insufficient information. What were the precise differences sought with standard deviations, effect sizes, etc.? ... These calculations should be checked against a more standard clinical trials sample size software ...”

Response:

Thank you for this important observation. We have now added detailed information about the sample size calculation to the Methods section. In the reference study, the mean change in the treatment group was 0.877 ± 0.799 (mean±SD), while in the control group it was -0.279 ± 0.729 (mean±SD). Based on these data, the calculated Cohen’s d effect size was approximately 1.51. Using G*Power with 95% power (power = 0.95) and a 5% significance level (α = 0.05), the estimated required sample size was 11 participants per group. To account for an anticipated dropout rate of approximately 25%, we planned to recruit additional participants. The study was ultimately completed with a total of 28 participants.

This clarification has been added to the Sample Size Calculation subsection of the Methods.

2. Parametric vs. non-parametric tests and normality assumption

Reviewer comment:

“The Statistical Analysis section refers to normal tests. However, the Tables presented look to be done non parametrically. If normality was assumed ...”

Response:

Thank you for pointing this out. We performed normality testing (Shapiro–Wilk) on our data and found that the assumptions for parametric tests were not satisfied. Therefore, non-parametric tests were used throughout the analysis. This choice is now clearly described in the Statistical Analysis section.

3. Multiple comparisons adjustment

Reviewer comment:

“Tables 2 to 4 show many statistical comparisons ... p-value adjustment for multiple comparisons should be examined ...”

Response:

We appreciate this comment. In our study, the main comparisons involved two independent groups. Since there were no multiple groups or multiple factors tested simultaneously, corrections such as Bonferroni adjustment were not deemed necessary.

**Reviewer #2:**

Dear Reviewer,

Thank you for your detailed review and constructive feedback on our study. Below, we provide our point-by-point responses to each of your comments:

1. Reviewer comment:

It would be useful to include some important demographic details in this section, such as gender distribution, stroke etiology (ischemic/hemorrhagic), and affected side (right/left).

Response:

The requested information has been provided in Table 1.

2. Reviewer comment:

However, some expressions (for example: “the therapist gently pulls the arm and trunk backwards…”) need clarification in terms of anatomy and biomechanics.

Response:

The noted ambiguity has been resolved, and the text has been revised for clarity.

3. Reviewer comment:

The extent to which the procedure was effective in terms of blinding (e.g., whether participants guessed their group) is not specified.

Response:

It has been clarified that the technique applied to patients was presented as a therapeutic approach, and no participant realized they were receiving a sham procedure.

4. Reviewer comment:

More information on session length and degree of individualization in content for NDT would be helpful.

Response:

The NDT procedure has been described in detail.

5. Reviewer comment:

It is not stated how many different assessors made the assessments and whether the assessors were blinded.

Response:

No blinding was performed. The study was designed and conducted as a randomized controlled trial, and all assessments were carried out by a single researcher.

6. Reviewer comment:

It would have been appropriate to refer to the validity and reliability information of the device.

Response:

Several reference articles regarding the use of the device in stroke patients have been provided.

7. Reviewer comment:

Taking the effect size as 1 is a very high assumption. It should be stated which source or preliminary study this value is based on.

Response:

In the reference study, the mean (±SD) changes were reported as 0.877 ± 0.799 in the treatment group and -0.279 ± 0.729 in the control group. Based on these data, the calculated Cohen’s d effect size was approximately 1.51. Using G*Power with 95% power (power = 0.95) and a 5% significance level (α = 0.05), the estimated required sample size was 11 participants per group. To account for an anticipated ~25% dropout rate, we planned to recruit 14 participants per group. The study was ultimately completed with a total of 28 participants.

8. Reviewer comment:

Sentence structures should be simplified; especially the part explaining the GFM application should be written in clearer and more technical terms.

Response:

Revisions have been made to address these ambiguities and improve clarity.

9. Reviewer comment:

A reference to the effect size used in the sample size calculation should be provided.

Response:

The reference used for the sample size calculation has been added to the manuscript.

10. Reviewer comment:

It should be stated whether the person(s) performing the spasticity assessment are the same person and whether blindness is ensured.

Response:

No blinding was performed. The study was designed and conducted as a randomized controlled trial, and all assessments were carried out by a single researcher.

11. Reviewer comment:

There are grammar and fluency problems in some sentences. Example: In the sentence “Participants in both groups had similar in terms of…” the expression “had similar” is used incompletely. The correct usage should be “were similar in terms of…”.

Response:

Thank you for pointing this out. The relevant sentences have been revised accordingly.

**Reviewer #3:**

Dear Reviewer,

Thank you very much for your thorough and constructive comments on our manuscript. We have carefully considered each point and revised the text accordingly. Below, we provide point-by-point responses to all of your suggestions:

1. Reviewer comment:

Consider adding "stroke patients" to the keywords for better indexing. The final sentence about long-term outcomes could be omitted in the abstract (due to word limits) and moved to the conclusion section of the main text.

Response:

The phrase “stroke patients” has been added to the keywords. However, to emphasize the importance of this aspect, the phrase “long-term outcomes” was intentionally retained in the abstract.

2. Reviewer comment:

The hypothesis (H1) should be rephrased in academic style, e.g., “It was hypothesized that functional massage would significantly improve...”.

Response:

The hypothesis statement has been revised as suggested to follow academic style.

3. Reviewer comment:

Massage description is written in an active, first-person tone (“The therapist stands…”). It should be revised into passive academic tone, e.g., “The therapist was positioned behind the patient…”. "10 GFM sham application" likely contains a typo — should be “10 minutes of sham GFM”. MMSE cut-off is listed as 23 — typically, 24 is used. Clarify with reference.

Response:

The relevant section has been rewritten in passive voice for academic tone. Information on the duration of GFM (10 minutes) has been added. Regarding MMSE, it was intended to indicate scores greater than 23, but was mistakenly written as “at least 23.” The text has been corrected to specify “at least 24.”

4. Reviewer comment:

Verify consistency between text and tables (e.g., changes in cadence or stride parameters).

Response:

Values for cadence and stride parameters have been checked for consistency between the text and the tables.

5. Reviewer comment:

Blinding details are not discussed. Was this single-blind or double-blind?

Response:

The relevant section has been reviewed, and the absence of blinding has been explicitly added as a limitation.

6. Reviewer comment:

Several minor spelling errors (e.g., “muslce” → “muscle”, “spacticity” → “spasticity”). Avoid direct instructions or first-person tone in the methods section.

Response:

All identified spelling errors have been corrected. The relevant sections have been revised to use passive academic language throughout.

**Reviewer #4:**

Dear Reviewer,

Thank you very much for your thorough and constructive review of our manuscript. We appreciate the time and effort you dedicated to providing detailed feedback. Below, we address each of your comments point by point.

1. Reviewer comment:

The manuscript aims to highlight the beneficial effects of functional massage applied in association with neurodevelopmental treatment...

Response:

Thank you for your overall positive evaluation and for recognizing the novelty and clinical value of our approach.

2. Reviewer comment:

The title effectively captures the core elements of the study; however, while informative, it is somewhat lengthy and includes a minor inconsistency. Throughout the article, the term "functional massage" is consistently used, whereas the title refers to "function massage."

Response:

Thank you for your careful attention. To eliminate this inconsistency and avoid confusion, we have revised the title to consistently use the phrase “functional massage.”

3. Reviewer comment:

While the introduction reflects a comprehensive understanding of the clinical dimensions... The theoretical foundation remains insufficiently supported...

Response:

We appreciate this valuable observation. We revised the Introduction section to more clearly and thoroughly explain the neurophysiological effects of functional massage. This enhancement improves the clarity and readability of the section and provides a stronger theoretical foundation for the study.

4. Reviewer comment:

It would be useful to clarify the exact time frame: whether each participant received treatment for 6 weeks within this extended period (May–November), or whether the stated period reflects the total duration of the intervention for all participants.

Response:

We have clarified that each participant received treatment for 6 weeks within the overall study period.

5. Reviewer comment:

The paragraph contains noticeable redundancy, particularly in the repeated use of the term "spasticity."

Response:

We have revised the paragraph to reduce repetition, shortened the content to improve clarity, and limited the use of the term “spasticity” for better readability.

6. Reviewer comment:

Please check out the "doi" from Reference nr. 15...

Response:

The incorrect DOI has been corrected to ensure accurate citation.
