# Supplementary material for: Effects of gastrocnemius functional massage on lower extemity spasticity, spatio- temporal gait variables and fall risk in patients with stroke: A randomized controlled trial
Source: PLoS One. 2025 Sep 24;20(9):e0332308. doi: 10.1371/journal.pone.0332308 (PMC12459846; doi:10.1371/journal.pone.0332308)
Supplement: Attachment — Submitted filename: Response_to_Reviewers_auresp_2.docx [file pone.0332308.s007.docx]

Dear Academic Editor,

We would like to express our sincere gratitude for the constructive evaluations and suggestions provided. We have carefully reviewed the valuable comments of the reviewers and revised our manuscript accordingly. We believe that these revisions have improved the clarity and scientific quality of our work. Below, we provide our detailed responses to each reviewer’s comments, preserving their original wording.

**Reviewer #1:** One can see from the results section that the analyses showed significant changes in the EG group and mostly no significant changes in the CG subjects. The authors should thus tell the reader why no direct p-value was calculated or displayed in the Tables 2 to 4 comparing the pre to post changes in CG vs EG for each result. This could be a limitation of the study and require a larger future trial requiring a more adequate sample size. The Discussion section should be expanded accordingly.

**Response:** We thank the reviewer for this valuable comment. In fact, p-values for both intra-group and inter-group comparisons were calculated and were already presented in Tables 2–4. To make this distinction clearer for readers, we have now added superscript letters a and b next to the p-values, where:

pᵃ: Intergroup comparison

pᵇ: Intragroup comparison

This explanation has been added below the tables to ensure that the distinction between intra-group and inter-group comparisons is clearly understood.

**Reviewer #2:** I would like to inform you that I found the article you submitted to be scientifically sufficient. Your topic selection, methodological approach, and the contributions of your findings to the field were noteworthy. I believe your work will make valuable contributions to the literature. I thank you for your efforts and wish you continued success.

**Response:** We are grateful for your positive feedback and encouraging words. Your appreciation of our methodological approach and the potential contribution of our findings is highly motivating for our research team.

**Reviewer #3:** The revisions have been reviewed and the article has been deemed appropriate. I think it is suitable for publication. you.

**Response:** We thank you for your positive assessment and for recognizing the adequacy of the revised manuscript. Your supportive comments are greatly appreciated.

**Reviewer #4:** I appreciate the authors' effort to respond rigorously and seriously to the reviewers' comments. All the issues raised have been adequately clarified, including strengthening the theoretical basis in the introduction, standardizing terminology, and detailing the duration of the intervention. The revised version of the manuscript brings significant improvements in terms of clarity, coherence, and scientific quality of the paper. I congratulate the authors for the careful way in which they integrated the feedback and for the obvious concern to perfect the study.

**Response:** We greatly appreciate your recognition of the improvements made in the revised manuscript. Your constructive feedback in the earlier review round was invaluable in enhancing the clarity, coherence, and scientific quality of our work.
